# NanoARG: a web service for detecting and contextualizing antimicrobial resistance genes from nanopore-derived metagenomes

**DOI:** 10.1186/s40168-019-0703-9

**Published:** 2019-06-07

**Authors:** G. A. Arango-Argoty, D. Dai, A. Pruden, P. Vikesland, L. S. Heath, L. Zhang

**Affiliations:** 1 Department of Computer Science, Virginia Tech, Blacksburg, VA USA; 2 Department of Civil and Environmental Engineering, Virginia Tech, Blacksburg, VA USA

**Keywords:** Metagenomics, Nanopore sequencing, Antibiotic resistance, Metal resistance, Mobile genetic elements

## Abstract

**Background:**

Direct and indirect selection pressures imposed by antibiotics and co-selective agents and horizontal gene transfer are fundamental drivers of the evolution and spread of antibiotic resistance. Therefore, effective environmental monitoring tools should ideally capture not only antibiotic resistance genes (ARGs), but also mobile genetic elements (MGEs) and indicators of co-selective forces, such as metal resistance genes (MRGs). A major challenge towards characterizing the potential human health risk of antibiotic resistance is the ability to identify ARG-carrying microorganisms, of which human pathogens are arguably of greatest risk. Historically, short reads produced by next-generation sequencing technologies have hampered confidence in assemblies for achieving these purposes.

**Results:**

Here, we introduce NanoARG, an online computational resource that takes advantage of the long reads produced by nanopore sequencing technology. Specifically, long nanopore reads enable identification of ARGs in the context of relevant neighboring genes, thus providing valuable insight into mobility, co-selection, and pathogenicity. NanoARG was applied to study a variety of nanopore sequencing data to demonstrate its functionality. NanoARG was further validated through characterizing its ability to correctly identify ARGs in sequences of varying lengths and a range of sequencing error rates.

**Conclusions:**

NanoARG allows users to upload sequence data online and provides various means to analyze and visualize the data, including quantitative and simultaneous profiling of ARGs, MRGs, MGEs, and putative pathogens. A user-friendly interface allows users the analysis of long DNA sequences (including assembled contigs), facilitating data processing, analysis, and visualization. NanoARG is publicly available and freely accessible at https://bench.cs.vt.edu/nanoarg.

**Electronic supplementary material:**

The online version of this article (10.1186/s40168-019-0703-9) contains supplementary material, which is available to authorized users.

## Background

Antimicrobial resistance (AMR) compromises the ability to prevent and treat infectious disease and represents a highly significant and growing global public health threat [1]. It is currently estimated that the annual number of deaths worldwide due to antibiotic resistance will top ten million by 2050 [2]. In response, numerous national and international agencies have called for expanded monitoring both in the clinic as well as in environmental settings. In particular, environmental monitoring can provide insight into not only human and agricultural inputs of antibiotic-resistant bacteria and antibiotic resistance genes (ARGs), but also factors contributing to the evolution and spread of resistant pathogens. For instance, various environmental compartments, such as wastewater treatment plants, livestock lagoons, and amended soils, can act as “environmental reactors,” in which resistant bacteria discharged from domestic, hospital, industrial, and agricultural waste streams have the opportunity to interact with native aquatic and soil bacteria in the presence of selection pressures to potentially give rise to new resistant forms [3, 4]. Humans may subsequently be exposed to resistant organisms via consumption of food crops affected by biological soil amendment or irrigation, as well as through contact with treated and untreated water used for recreational, hygienic, and potable purposes [5, 6].

Molecular-based monitoring presents many advantages over culture-based techniques for tracking antibiotic resistance in the environment. This is particularly true with respect to the potential to recover rich information regarding the carriage and movement of ARGs within complex microbial communities. Culture-based techniques are time-consuming and only provide information about one target species at a time, thus potentially overlooking key microbial ecological processes that contribute to the spread of AMR. Thus, directly targeting ARGs as “contaminants” of concern that transcend bacterial hosts has gained popularity. In particular, horizontal gene transfer (HGT) [7] plays a critical role in the rise of new resistant strains and the dissemination of AMR in microbial ecosystems [8]. Intercellular transfer of ARGs among bacteria is facilitated via mobile genetic elements (MGEs), such as transposons, plasmids, and integrons [9]. Integrons are key genetic elements of interest as they facilitate capture of multiple ARGs, thus effectively functioning as vehicles for dissemination of multidrug resistance [10]. The mechanisms involved in HGT include conjugation, transformation, transduction, and homologous recombination, where DNA is incorporated by transposition, replication, and integration [9].

Multidrug resistance has emerged as a major clinical challenge. For example, methicillin-resistant *Staphylococcus aureus* (MRSA) is responsible for major hospital infections, with few options for treatment, especially when resistant to vancomycin [11]. More recently, New Delhi Metallo beta lactamase (*bla*NDM-1) has emerged as a major concern, as it encodes for resistance to powerful last-resort carbapenem antibiotics and is carried on a highly mobile genetic element associated with multidrug resistance that has been detected in several different pathogenic species, including *Escherichia coli*, *Klebsiella pneumoniae*, *Providencia rettgeri*, and *Acinetobacter baumannii* [12–14]. This example emphasizes that, ideally, monitoring technologies should provide a rapid and robust characterization of ARGs and their likely association with MGEs, multidrug resistance, and carriage by pathogen hosts. In this regard, shotgun metagenomic sequencing techniques have emerged as a promising tool for the characterization of the diverse array of ARGs found in different environments [4, 15–17]. In particular, high-throughput next-generation DNA sequencing technologies, such as the Illumina platform [18] and 454 pyrosequencing [19, 20], have enabled a new dimension to ARG monitoring in the environment.

While providing unprecedented amounts of sequence information (360,081 metagenomes processed on MG-RAST [21], 20,120 on EBI-metagenomics [22], and 3038 on MetaStorm [23]), a major drawback of these technologies is the very short DNA sequence reads produced, at most a few hundred nucleotides long. Nonetheless, next-generation DNA sequencing is growing in use as a powerful means of profiling ARG occurrence in various environments. ARGs can be identified by direct annotation through comparing sequences against available ARG databases. This enables relatively quantitative comparisons, including relative abundance calculations (e.g., normalization to 16S rRNA genes or total ARGs). Alternatively, short reads can be assembled into longer contigs for assembly-based annotation, which can improve resolution in identifying ARGs and can also provide information about neighboring genes. Both approaches have limitations. The first can only be used to detect previously described ARGs that populate available databases [24] and requires determination of an arbitrary DNA sequence identity cutoff [25]. This process generally undermines the possibility to identify novel ARGs, although a novel similarity-based method was recently proposed to annotate ARGs with low similarity to existing database ARGs [26]. Assembly, on the other hand, requires deeper and more costly sequencing along with greater computational resources [27] and still can produce incorrect contigs and chimeric assemblies [28]. For these reasons, it is important to be cautious in interpreting results derived from the assembly of short sequence reads because of the possibility of assembly errors and the lack of standard means to estimate confidence in assembly accuracy [29–31]. Also, quantitative value of data is lost following assembly.

In 2014, Oxford Nanopore Technologies (ONT) released the MinION nanopore sequencer, which provides long sequence reads averaging 5 kb in length [32] and even upwards of 100 kb [33]. A major disadvantage of nanopore technology, however, is the high error rate, estimated by Jain et al. to be below 8% [34]. However, this error rate represents a marked improvement over an earlier estimated error rate of 38% [35], with a general trend towards reduced error rates with the help of read correction algorithms [36]. It has been shown that nanopore technology can produce highly accurate assemblies, in the range of 95% when applied to whole-genome sequencing [37–39]. Nanopore sequencing has also been applied for shotgun metagenomics, including identification of viral pathogens [40], assessment of microbial diversity in extreme environments [41], and detection of ARGs in various environments [42–47]. To date, nanopore sequencing has not been applied for the purpose of metagenomic profiling of ARGs in environmental samples.

Long nanopore reads offer a unique opportunity to explore the context of ARGs in terms of co-occurrence and potential for mobility. Unlike de novo assembly of short reads into longer contigs that might produce chimeric sequences [48], nanopore sequencing inherently yields long sequences, thus reducing the potential for chimeras. Therefore, nanopore sequencing has potential to become a powerful tool for the identification of the coexistence of ARGs, MGEs, and MRGs. Such an approach could substantially advance environmental monitoring approaches, providing insight into the potential dissemination of AMR through co-occurrence and co-selection of ARGs and other relevant genes and genetic elements [49–51]. The co-occurrence of ARGs and MGEs also enables tracking of evidence of genetic events of interest, such as HGT [46].

Here, we introduce NanoARG, a user-friendly online platform that enables comprehensive profiling of ARGs in environmental samples using nanopore sequencing data. In addition to comprehensive ARG profiling, NanoARG also provides identification of MRGs, MGEs, taxonomic markers, and sequences with high similarity to known pathogens, along with interactive visualization of linkages among these various elements on the same DNA strand. To demonstrate the potential of NanoARG for environmental ARG profiling, several nanopore sequencing libraries, including environmental and clinical samples, were analyzed. The Web service is freely available at https://bench.cs.vt.edu/nanoarg. It requires a user login and subscription to upload and process nanopore sequencing data.

## Implementation

### Web service and pipeline

**Fig. 1:**
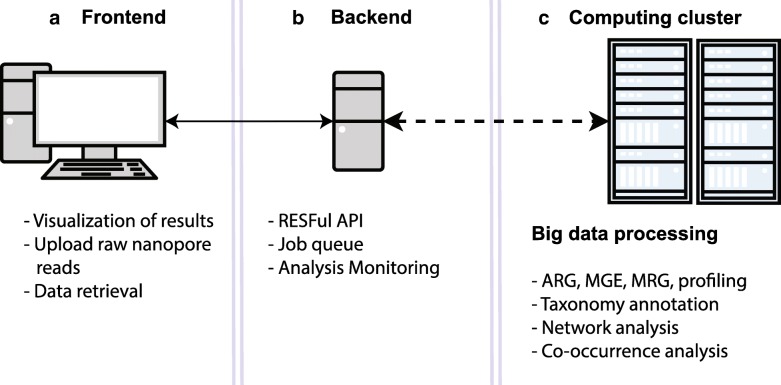
NanoARG architecture. **a** The front end is the link between users and the analytical tools, allowing raw data upload and result visualization. **b** A back end RESTful API manages the data, triggers the analysis, and monitors the status of the analysis. **c** The computing cluster module processes the data and executes ARG, MGE, MRG, and taxonomic profiling

**Fig. 2:**
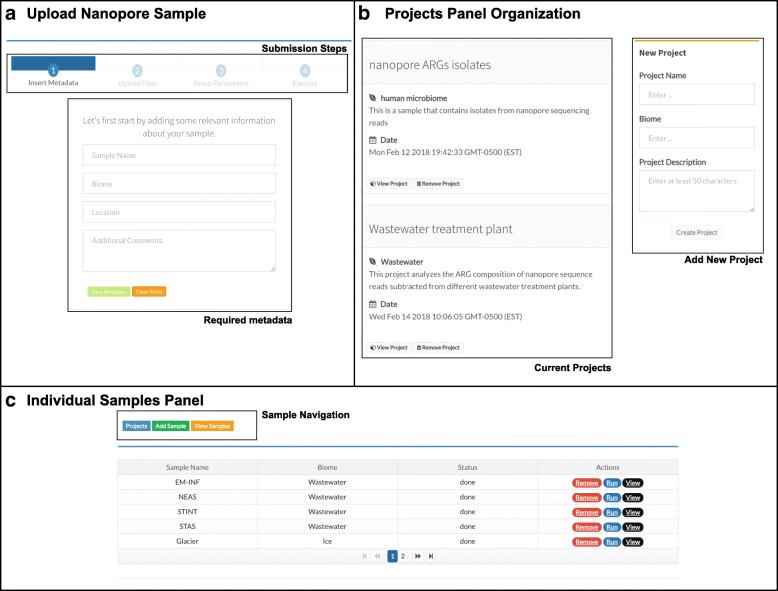
User interface. **a** Steps and metadata required to upload samples to NanoARG. **b** Projects are organized based on the creation date and visualized as a timeline post. **c** List of samples under a project displaying basic metadata (biome), the monitor variable (status), and the three actions that can be performed by users

Figure 1 illustrates the NanoARG architecture. The workflow has three major components: (1) a web interface, where users can upload data and monitor the progress of the analysis (Fig. 1a); (2) a Representational State Transfer (RESTful) application program interface (API), which monitors and sends the raw MinION nanopore sequencing data to a computing cluster for processing (Fig. 1b); and (3) a back end platform for retrieval of results and downstream analyses (Fig. 1c), such as taxonomic annotation, gene co-occurrence analysis, human pathogen-like sequence detection, network analysis, and multiple sample comparisons. The nanopore reads are screened against databases currently available using different ‘omics tools, both of which will be updated in the future when an improved version is available. Results are stored as JavaScript Object Notation (JSON) files. Metadata and user information are encrypted and stored in a Mongo database. The workflow runs on a large distributed system in the Advanced Research Computing (ARC) center at Virginia Tech. The cluster is managed by the qsub queuing system [52].

The Web service provided by NanoARG includes several features to facilitate analysis of environmentally derived metagenomic data obtained via nanopore sequencing. Users can submit data to the NanoARG Web service using a simple graphical user interface (Fig. 2a). In the current version of NanoARG, data submitted to the system is stored privately. To start using the service, users are required to register an account with their email address, which allows them to manage and control submitted samples and projects. Users can voluntarily share their projects with other users by sharing additional email addresses. To create a project, a few parameters, such as name, description, and biome type (Fig. 2b), are required. Inside each project, users can add new samples, run new analyses, or remove or rerun existing samples (Fig. 2c).

NanoARG provides several types of visualizations to interpret the results and allows users to download results (e.g., absolute and relative abundances, co-occurrence network associations, taxonomy annotations, and ARG context patterns) in a tabular format containing the fields required for tuning the results (*E*-value, identity percentage, and coverage). These tables can be used for further processing and statistical analysis. The NanoARG website was developed using the Google Angular 5 framework (https://angular.io), the back end was developed under the Node.js framework (https://nodejs.org/en/). Finally, the computing pipeline was developed using the Luigi framework, allowing the monitoring and rescheduling of jobs that failed during execution (https://github.com/spotify/luigi).

### Required data types

NanoARG requires users to upload nanopore reads in FASTA format [53], thus requiring that the users have already preprocessed the raw fast5 files from the nanopore sequencing device. This step can be done using a base-calling program such as Albacore [54], Metrichor [32], or Nanocall [55], with a sequence extractor toolkit such as poretools [56]. Barcode recognition and read sorting by barcodes can be conducted along with base calling. Before submitting data to the system, users must provide simple metadata consisting of sample name, biome, location, and comments and can also manually enter details about DNA extraction methodology, if so desired. Then, following four simple steps (insert metadata, upload files, set up parameters, and execute), users can submit the data and initiate analysis (Fig. 2a).

### Data processing

**Fig. 3:**
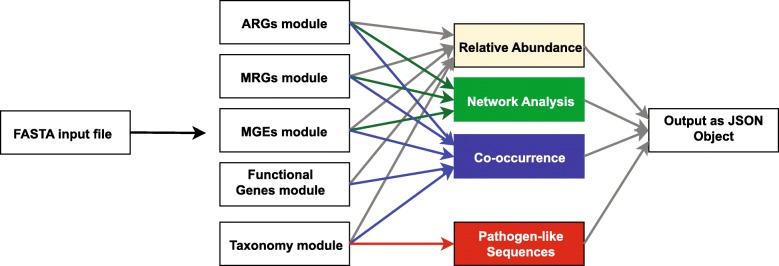
General overview of the NanoARG pipeline. FASTA input reads are processed by five modules to annotate reads according to ARGs, MRGs, MGEs, and other functional genes and taxonomic affiliation. Annotations are then processed through several stages to achieve the desired analysis (relative abundance, network analysis, co-occurrence, and putative pathogens). All analyses are packed into a JavaScript Object Notation (JSON) file that can be easily streamed using an http request

Once the data is uploaded to the computing cluster, it is processed by several modules that perform a set of tasks to obtain annotation profiles for ARGs, MGEs, MRGs, and associated taxa (Fig. 3). The status of the analysis can be easily monitored through the user interface (Fig. 2c).

### Clustering of local best hits for annotating ARGs, MRGs, and MGEs

**Fig. 4:**
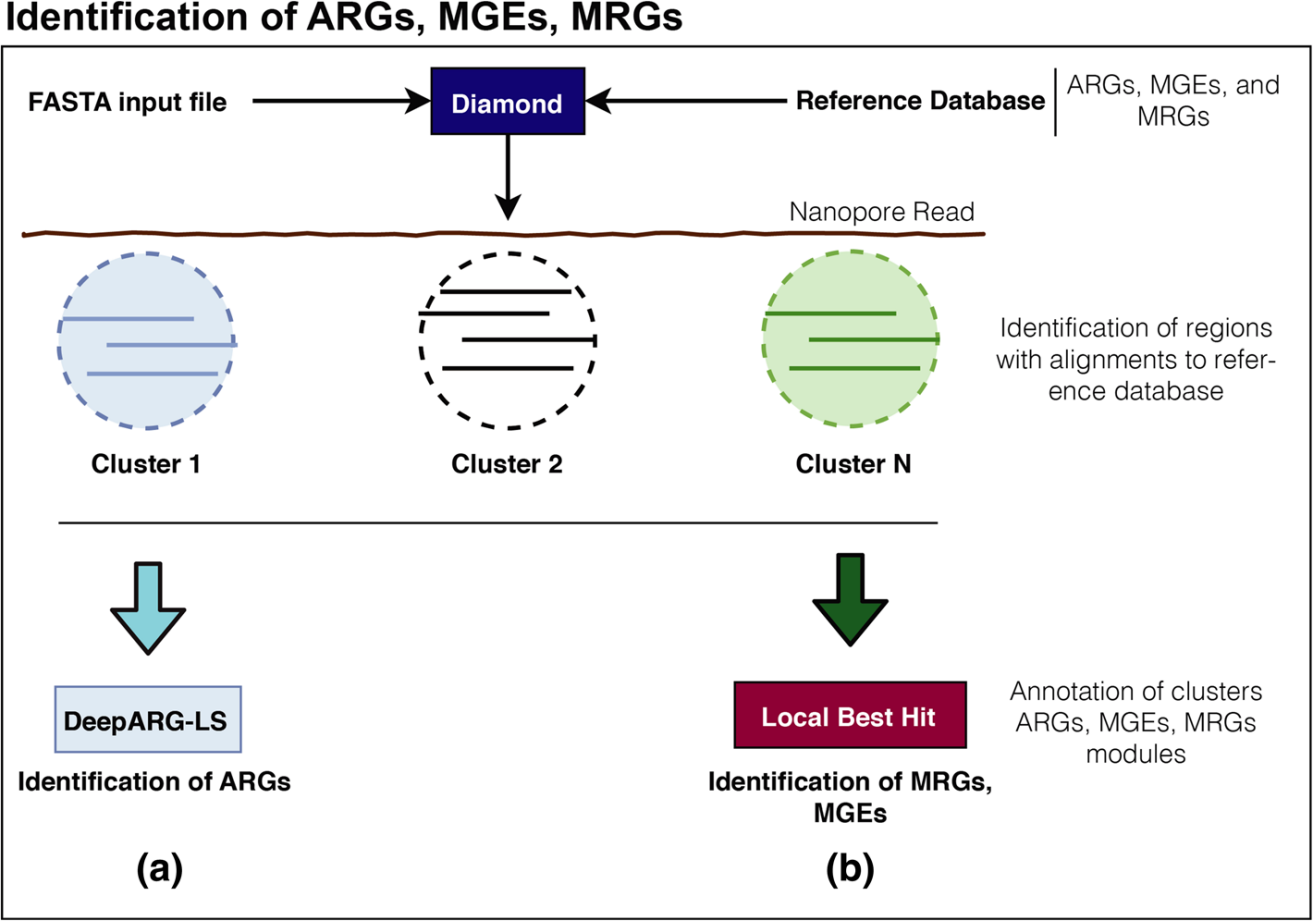
Annotation pipelines. **a** Identification of ARGs: input nanopore reads are aligned to the DeepARG database using DIAMOND. Alignments are clustered based on their location and annotations are performed using the DeepARG-LS model. **b** Local Best Hit Approach: identification of the functional genes within the nanopore reads. Alignments are clustered based on their location and the best hit for each cluster is selected. Resulting alignments are filtered out based on sequence alignment quality

Traditionally, the analysis of long sequence reads, such as assembled contigs, is achieved by first identifying open reading frames (ORFs) within the sequences [23, 57–59] and then searching (e.g., by utilizing BLAST) the ORFs against a database for functional annotation. While nanopore sequences are analogous to long contigs, the high sequencing error rate can limit detection of ORFs. Therefore, NanoARG deploys DIAMOND [60] to align reads against the corresponding databases. Then, it clusters all the local best hits into regions and determines the annotation of each region using either the best hit approach or the DeepARG prediction [26], as shown in Fig. 4. Specifically, DIAMOND [60] is run with permissive parameters (*E*-value 1e−5, identity 25%, coverage 40%, and --nk 15000), while bedtools [61] is used to cluster the local best hits in each read into regions. Table 1 describes the databases, methods, and parameters used in NanoARG. The resulting regions/clusters are then annotated for ARGs, MRGs, and MGEs, as detailed below.

**Table 1:** NanoARG modules, parameters, and methods

Module	Database	Method	Parameters
ARGs	DeepARG-db	DeepARG-LS	--iden 25 --prob 0.5 --cov 0.4
MGEs	NCBI-NR + I-VIP	Diamond	--evalue 1e-5 --iden 25 --nk 15000
MRGs	BacMet	Diamond	--evalue 1e-5 --iden 25 --nk 15000
Taxonomy	Bacteria, Aarchaea, Viruses, Human	Centrifuge	default
Pathogens	ESKAPE + WHO	Pattern matching to NCBI Taxa ID	–NA

### ARG module

Following the clustering procedure of the local best hits to identify putative regions of interest (Fig. 4), NanoARG uses the DeepARG-LS model, a novel deep learning approach developed by Arango-Argoty et al. [26] to detect and quantify ARGs within the regions. A fundamental advantage of the DeepARG model is its ability to recognize ARG-like sequences without requiring high sequence identity cutoffs, which is especially useful for nanopore sequences with high sequencing error rates. The DeepARG-LS model is applied with permissive parameters, specifically, an identity cutoff of 25%, a coverage of 40%, and a probability of 0.5, to predict that a region corresponds to an ARG.

Abundance of ARG classes and groups is estimated by the copy number of ARGs. To enable comparison of ARG abundance across samples, analogous to the approach described by Ma et al. [58], the copy number of ARGs is normalized to the total gigabase pairs (Gbp) of the sample to obtain the relative ARG abundances:

$$ {A}_i=\frac{C_i}{C_g} $$(1),

where *C*_*i*_ corresponds to the total count of ARG *i* (copies of the ARG) and *C*_*g*_ corresponds to the size of the data set in Gbp, that is, *C*_*g*_ = *Γ*/*μ*_*g*_, where *Γ* is the total number of nucleotides in the library and *μ*_*g*_ = 1 × 10^9^ corresponds to 1 Gbp.

### MRG module

To annotate MRGs, NanoARG queries the BacMet database [62]. Following clustering of the local best hits to identify putative regions of interest (Fig. 4), NanoARG identifies and categorizes clusters to MRGs according to their best hits. Absolute (copy number) and relative abundances of MRGs are computed using **Eq. (1)**.

### MGE database and annotation module

MGEs were identified from the National Center for Biotechnology Information (NCBI) non-redundant database by using a keyword search [63]. Thus, genes related to any of the following keywords—transposase, transposon, integrase, integron, and recombinase—were labeled as associated MGEs. In addition, a set of integrases and class 1 integrons (*Int*I1) were added from the integron-integrase (I-VIP) database [64]. All sequences were clustered using CD-HIT [65] with an identity of 90%. The resulting MGE database consists of 227,640 genes. Similar to the annotation strategy adopted for MRGs, nanopore reads are annotated using the MGE database and relative abundance of MGEs is computed using **Eq. (1)**.

### Taxonomic annotation module

Nanopore reads are classified according to taxonomic lineage using Centrifuge [66], a fast and accurate metagenomic classifier that uses the Burrows-Wheeler transform (BWT) and FM-index. Centrifuge is executed with default parameters (--min-hitlen 25 -f -k 50). Taxonomic relative abundance is estimated by Centrifuge using an expectation maximization (EM) algorithm similar to the one used in Cufflinks [67] and Sailfish [68]. This allows the abundance estimation to be sensitive to genomes that share nearly identical genomic regions. Therefore, each nanopore read is assigned to a particular taxonomic lineage. In addition, nanopore reads not successfully processed by Centrifuge were labeled as unknown.

### Co-occurrence of ARGs, MGEs, and MRGs

To support users in exploring the co-occurrence of ARGs, MGEs, and MRGs in nanopore data sets, NanoARG reports all reads that contain at least one ARG, along with its neighboring genes. This data is presented in a tabular format, where each entry contains the start position, end position, gene coverage, percent identity, *e*-value, strand (forward or reverse), and taxa corresponding to each read. Furthermore, NanoARG provides a gene map that depicts the gene arrangement, which is useful for visualizing the gene’s co-occurrence and context. Overall co-occurrence patterns are depicted as a network, where nodes represent genes, node sizes represent the number of occurrences, edges between nodes represent genes’ co-occurrence, and edge thickness depicts the number of times the co-occurrence pattern is observed in the data set. Links among nodes are added according to their co-occurrence among the nanopore reads. The network is rendered using cytoscape.js [69].

### World Health Organization priority pathogens

The World Health Organization published a list of pathogens that are of particular concern with respect to the spread of antimicrobial resistance [70]. This list consists of three priority tiers, namely, critical, high, and medium, as described in Table 2. Similarly, the ESKAPE database houses multidrug-resistant pathogens that are critical to human health [71]. These two resources are employed by NanoARG to identify the potential presence of critical pathogens in sequenced samples. Briefly, nanopore reads are matched against sequences available for critical pathogens by examining the NCBI taxonomic identifier downloaded from the NCBI taxonomy website. Note that NanoARG refers to these hits as “potential” pathogens because the presence of true pathogens cannot be confirmed without higher resolution methods, such as whole genome sequencing and viability confirmation.

**Table 2:** Twelve species of pathogenic bacteria prioritized by the World Health Organization (WHO) as representing substantial antibiotic resistance concern. WHO classification is based on the three categories according to the impact on human health and need for new antibiotic treatments

Importance	Pathogen	Confer resitance to
Critical	*Acinetobacter baumannii*	Carbapenem
	*Pseudomonas aeruginosa*	Carbapenem
	*Enterobacteriaceae*	Carbapenem, ESBL-producing
High	*Enterococcus faecium*	Vancomycin
	*Staphylococcus aureus*	Methicillin, vancomycin
	*Helicobacter pylori*	Clarithromycin
	*Campylobacter* spp.	Fluoroquinolone
	*Salmonellae*	Fluoroquinolone
	*Neisseria gonorrhoeae*	Cephalosporin, fluoroquinolone
Medium	*Streptococcus pneumoniae*	Penicillin
	*Haemophilus influenzae*	Ampicillin
	*Shigella* spp.	Fluoroquinolone

### Application of NanoARG to nanopore sequencing datasets

To demonstrate NanoARG’s capability for profiling ARGs in the context of other relevant genes, four DNA extracts obtained from the influent sewage and activated sludge of three different wastewater treatment plants (WWTPs) were sequenced using the MinION nanopore sequencing platform and analyzed together with four publicly available nanopore metagenomic data sets using NanoARG (see Table 2 and Additional file 3).

### Nanopore sequencing of WWTP samples

Four WWTP samples (two influent sewage, two activated sludge) were collected from three WWTPs located in Hong Kong (HK_INF and HK_AS), Switzerland (CHE_INF), and India (IND_AS). Samples were preserved, transported, and subjected to DNA extraction using a FastDNA SPIN Kit for Soil (MP Biomedicals) as described by Li et al. [72]. DNA was purified with the Genomic DNA Clean & Concentrator kit (Zymo Research, Irvine, CA), and its concentration was quantified with the Qubit dsDNA HS Assay Kit (Thermo Fisher Scientific). DNA for each sample was pooled from triplicate extractions with equal mass. Pooled DNA was further purified and concentrated to meet the quality and quantity requirement for library preparation. The purity of DNA was then checked using a NanoPhotometer Pearl (Implen, Westlake Village, CA) via the two ratios of A260/280 and A230/260. Each DNA sample (1000 ng) was prepared individually for sequencing using the 1D Native Barcoding Genomic DNA kit (with EXP NBD103 & SQK-LSK108; Oxford Nanopore Technology) following the manufacturer’s protocol. Each sample was sequenced with a R9.4 flow cell for 24–48 h without local base calling. Sequence reads were base called using Albacore (v 1.2.4).

## Results and discussion

NanoARG is an online computational resource designed to process long DNA sequences for the purposes of annotating and co-locating ARGs, MGEs, and MRGs, and to identify their taxonomic hosts. Publication-ready figures and tables derived from these annotations can be directly produced, thus facilitating various dimensions of environmental monitoring and sample comparison.

### Visualization and data download

**Fig. 5:**
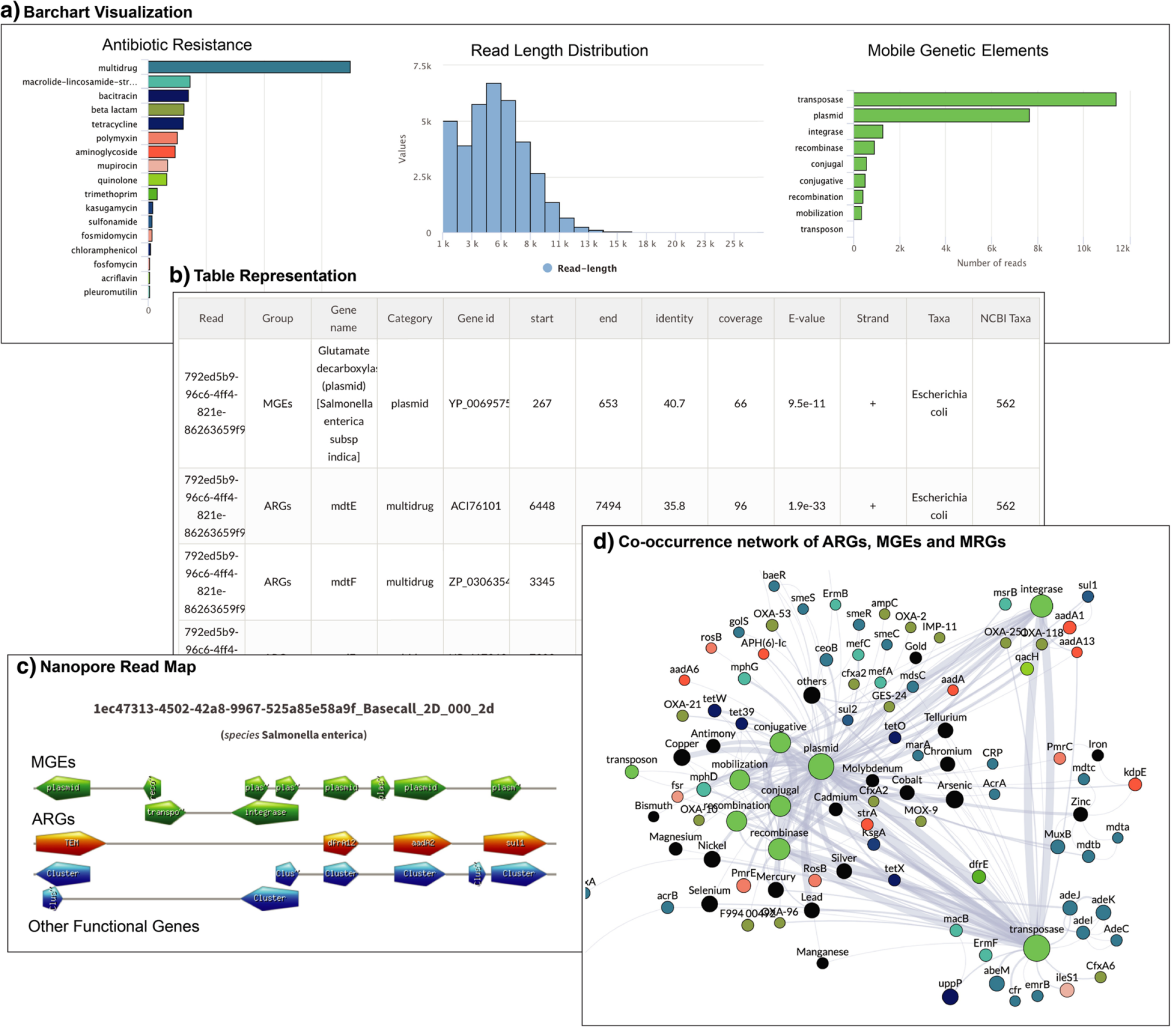
Visualization of NanoARG report. **a** Absolute abundances (read counts) are shown as bar charts as well as read length distribution and taxonomic counts. **b** Tabular data: results are also shown in tables containing all the relevant information for each annotation (*E*-value, coverage, identity, strand (forward, reverse), taxonomy, group, etc.). **c** Nanopore Read Map: this visualization organizes the gene matches in a linear format showing the co-occurrence patterns for each nanopore read with at least one ARG. **d** Co-occurrence Network of ARGs, MGEs, and MRGs: this interactive visualization allows users to drag and drop nodes to visualize the co-occurrence patterns in the sample

The NanoARG service provides a range of visualization options, including bar charts (Fig. 5a), tables (Fig. 5b), gene mapping charts (Fig. 5c), and co-occurrence networks (Fig. 5d) that display individual and combined analyses of ARGs, MGEs, and MRGs. Results can be downloaded from the tables and configured to include all data, without any filtering. This enables users to deploy their own filtering criteria and customize analyses.

### Effect of error correction in the detection of ARGs

To examine the effect of error correction in the detection of ARGs by NanoARG, HFS sample nanopore sequences were analyzed with and without error correction. The complete data set (library B) was downloaded from the poreFUME repository, including the raw nanopore reads (HFS-raw) along with the corrected reads after the poreFUME pipeline (HFS-poreFUME). In addition, the raw nanopore reads were also corrected (HFS-CANU) using the correction module from the CANU assembler. These three data sets were submitted to the NanoARG pipeline for annotation.

**Fig. 6:**
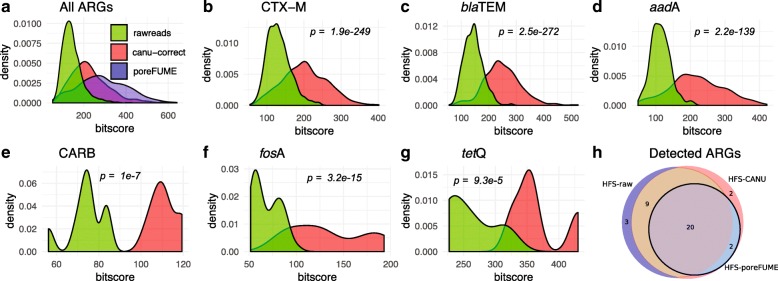
Comparison of error correction approach applied to a functional metagenomic sample. Comparison against raw reads and error-corrected reads using CANU correct and poreFUME. *p* values were computed between the different distributions using a *t* test. **a** Bit score distribution of all ARG alignments. **b**–**d** Comparison between raw and corrected reads using CANU correct for ARGs with high depth. **e**–**g** Bit score distribution for raw and corrected reads for low depth ARGs. **h** Venn diagram showing discovered ARGs by raw and corrected reads by CANU and poreFUME

Figure 6 a shows that the alignment bit score of all the ARGs is increased after read correction by both CANU and poreFUME algorithms compared to the raw uncorrected reads. Here, “high coverage” ARGs are those ARGs with ≥ 10 read hits whereas “low coverage” ARGs have fewer hits. For the CANU-correct algorithm, the bit scores of “high coverage” ARGs such as CTX-M, TEM, *aad*A, aac(6′)-I, and *erm*B ARGs were significantly improved (Fig. 6b–d) compared to the raw reads. Similarly, the bit scores of “low coverage” ARGs, such as CARB, *erm*F, *fos*A3, *mel*, and *tet*Q, also showed an improvement after read correction (Fig. 6e–g).

Figure 6 h depicts the intersection of ARG annotation by NanoARG among the three data sets (HFS-raw, HFS-CANU, HFS-poreFUME). ARGs with a minimum coverage of 80% and an identity greater than 30% were used for this comparison. Altogether, 22 unique ARGs were detected in the HFS-poreFUME data set, 32 in the HFS-raw data set, and 33 in the HFS-CANU data set. Out of the 22 ARGs detected in HFS-poreFUME, two ARGs (*abe*S and CARB) were not identified in the HFS-raw sample. Further examination revealed that these genes were actually detected in the HFS-raw data set but were removed after applying the filtering criteria described above. These two genes were also detected following the error correction step (HFS-CANU); indeed, all ARGs that were detected in HSF-poreFUME were also identified after applying the error correction algorithm with CANU. Although there were three uniquely identified ARGs in the HFS-raw data set (FosC2, LuxR, *emr*K) and four uniquely identified ARGs after CANU correction (CARB, OXY, *abe*S, *van*H), the results show that there was a transition in the annotation from raw to corrected reads. Thus, reads were reassigned to other ARGs with higher alignment and classification scores. For instance, raw reads containing the CTX-M gene were reassigned to the OXY gene with higher alignment scores in the HFS-CANU data set. The CARB gene was detected in both HFS-raw and HFS-CANU data sets. However, the coverage of this gene in the HFS-raw data set was below the 80% cutoff used for the analysis and therefore was removed from the list, whereas it was successfully detected in the HFS-CANU data set, showing an improvement in the alignment coverage. The reads containing the *fos*C2 gene in the HFS-raw sample were reassigned to the *fos*A gene in the HFS-CANU data set with higher alignment bit scores (73–126.3, respectively). Interestingly, the *van*H gene was detected exclusively on the HFS-CANU data set. These results show that the correction step enhances the detection of ARGs in MinION nanopore sequencing samples.

**Fig. 7:**
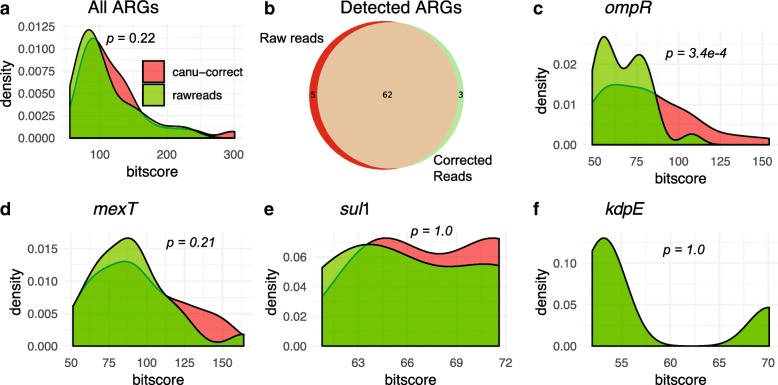
Effect of error correction on analysis of an environmental sample (WWTP influent). **a** Bit score distribution for all ARGs detected by NanoARG using the raw and CANU corrected reads. **b** Venn diagram showing the intersection of detected ARGs from raw and corrected reads. **c**–**d** Examples of the effect of correction in individual ARGs with high number of hits comparing the raw and corrected reads. **e**–**f** Effect of correction in ARGs with few hits from the raw and corrected data sets

To validate the read correction approach on a more complex sample than HFS, one WWTP sample (CHE_INF) subjected to direct shotgun metagenomic sequencing was selected for further validation of the effect of the error correction algorithm. The metagenomic data set was processed using CANU correct and submitted along with the raw data sets to NanoARG for annotation. poreFUME was not performed for this analysis because of dependency errors present during execution of the pipeline. Figure 7 a shows the bit score distribution of the ARG alignments for both raw and corrected reads. Notably, the correction algorithm did not significantly improve (*p* = 0.22) the overall ARGs bit score of the alignments for this more complex sample. Figure 7 b shows the intersection of the detected ARGs for the WWTP sample with and without correction. Among the majority of ARGs detected by NanoARG in both raw and corrected reads, three were detected after read correction, but not in the raw reads (OKP-A, *bcr*A, *otr*C). To observe the effect of coverage depth for each ARG, a closer examination of the individual ARGs did not indicate enhancement of alignment scores for genes with the greatest number of hits, such as *omp*R and *mex*T (Fig. 7c–d), or for ARGs with low numbers of hits, such as *sul*1 and *kdp*E (Fig. 7e–f). Because the overlap between the ARGs detected in the raw and corrected reads is greater than 95% (Fig. 7b), NanoARG was not further configured to perform error correction and lets users decide whether to upload raw, corrected reads, or assembled contigs. Users can find information about error correction and how to perform it using CANU on the NanoARG website.

The DeepARG-LS model deployed by NanoARG for ARG detection was extensively validated in its original development [26]. To further validate with respect to nanopore sequencing concerns, we examined the effect of a range of error rates (5%, 10%, 15%, 20%, 25%, and 30%) and read lengths (1 kb, 5 kb, 10 kb, 15 kb, and 20 kb) on ARG detection (see Additional file 3 for details). Our simulation results demonstrated that error rates had little effect on overall ARG detection (Additional file 3: Figure S1) and also on different ARG classes when each ARG class is considered separately (Additional file 3: Figure S2). The observation of high sequencing error rate having little effect on ARG detection was expected, given that DeepARG has been shown to be capable of identifying ARGs that have low sequence identity to known ARGs. For example, among 76 novel beta lactamase genes that had less than 40% identity to known beta lactamase genes and were discovered and verified experimentally [26], the DeepARG-LS model was able to identify 65 (= 85% accuracy, see [26] for details). Simulation results for read length indicate that the longer the read length, the more likely ARGs are detected (e.g., when read length reaches 10 kb or longer, more than 60% of the ARGs could be detected, see Additional file 3: Figure S3 for details). As the nanopore sequencing technology improves over time, longer reads are expected which will in turn benefit downstream sequence analyses.

To check the effect of time and consistency for the discovery of ARGs in nanopore samples using NanoARG, several data sets from the LSS sample were analyzed, including comparison of nanopore- versus Illumina-derived and whole-genome versus shot-gun data sets. Specifically, a study of lettuce spiked with *Salmonella enterica* (LSS) consisted of the following data sets: LSS-WGS (whole-genome sequencing), LSS-M (shotgun metagenomics), LSS-1.5hN (nanopore sequencing after 1.5 h), and LSS-48hN (nanopore sequencing after 48 h). To facilitate comparison, the short reads from LSS_WGS and LSS-M were first assembled using spades [31] with default parameters. Assembled scaffolds were subsequently submitted to NanoARG for annotation. The MinION nanopore sequencing libraries were first error corrected using CANU correct algorithm prior to submitting to NanoARG. To evaluate the accuracy of ARG detection, alignments were compared relative to a threshold identity cutoff greater than 80% and an alignment coverage greater than 90% from the LSS-WGS sample. A total 28 ARGs passed these filtering criteria, and further analyses were benchmarked against these 28 ARGs assuming a high level of confidence in their identity. Out of these 28 ARGs, two genes (*mdt*B and *bcr*) were not detected in the Illumina shotgun metagenomic dataset (LSS-M). When comparing the 28 benchmark ARGs set against the 1.5-h nanopore LSS-1.5hN sample, only four ARGs were detected (aac(6′)-I, *mdf*A, *mdt*G, *mdt*M) in the nanopore dataset. This result suggests that although nanopore sequencing offers a real-time alternative, the detection of specific ARGs would still require several hours. Still, when examining the 48-h nanopore sample (LSS-15hN), 25 out of the 28 benchmark ARGs were discovered. Interestingly, *mdt*B, one of the three undiscovered benchmark ARGs (*mdt*A, *mdt*B, and *mdt*C) from the LSS-48hN was not found by either the Illumina shotgun metagenomics sample (LSS-M) or the nanopore samples. These three ARGs were noted to pertain to the same antibiotic resistance mechanism. Overall, this analysis demonstrates general consistency of detection of ARGs in Illumina and nanopore sequencing libraries using NanoARG.

### Application of NanoARG to nanopore sequencing data

NanoARG provides users with a master table that contains the absolute and relative abundances of ARGs, MRGs, MGEs, and taxonomy annotations for each sample under a particular project. Relative abundances are computed as described in Eq. 1. Key attributes of this table are summarized in the following subsections, using eight nanopore sequencing data sets as examples.

### ARG abundance

**Fig. 8:**
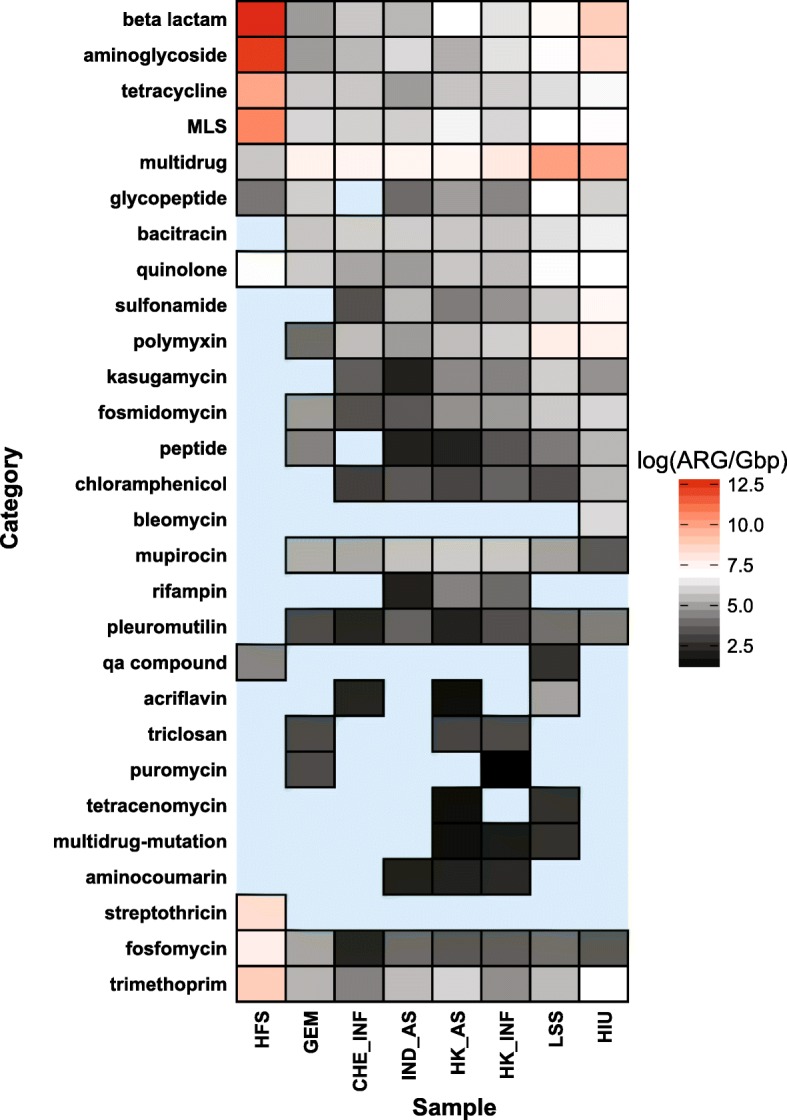
Relative abundance of antibiotic resistance classes for all biomes. Each cell in the heatmap corresponds to a particular antibiotic, biome pair. Color represents the copy number of ARGs divided by 1 Gbp on a logarithmic scale

WWTP samples contained the greatest number of reads (> 687,835), whereas human-derived samples (HIU, HFS) were comprised of much fewer reads (< 67,658) (See Table 3 for details). Figure 8 shows relative abundances of ARGs in the eight data sets. HFS contained the highest relative ARG abundance, likely due to the sample preparation approach that intentionally targeted genomic content associated with antibiotic resistance [73]. Comparatively, the direct shotgun metagenomic sequenced environmental samples had much lower ARG relative abundance. Among the WWTP samples, HK Influent and HK Effluent ranked the greatest in terms of relative abundance of ARGs.

**Table 3:** Sample collection, metadata, and total number of reads for all validation samples

Samples	Biome	Sample labels	Number of reads	Reference	Type of sample
Hong Kong activated sludge	Wastewater	HK_AS	3,307,368	This study	Complex microbial community
Hong Kong influent	Wastewater	HK_INF	2,724,813	This study	Complex microbial community
Switzerland influent	Wastewater	CHE_INF	687,835	This study	Complex microbial community
India activated sludge	Wastewater	IND_INF	1,925,639	This study	Complex microbial community
Artic glacier extreme metagenome	Glacier	GEM	344,966	Edwards, 2016	Complex microbial community
Heavily infected urine	Human associated	HIU	36,510	Schmidt, 2017	Enriched microbial community
Hospital fecal sample	Human associated	HFS	67,658	van der Helm, 2017	Enriched microbial community
Lettuce spiked *Salmonella*	Plant surface	LSS	211,806	Hyeon, 2018	Enriched microbial community

**Fig. 9:**
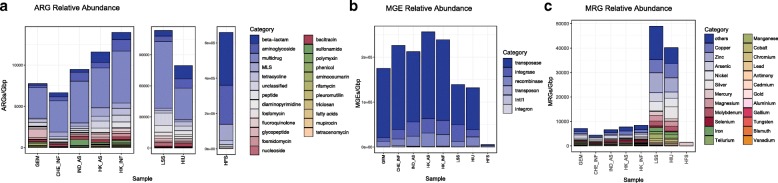
Relative abundance computed as copy of genes per 1Gpb of **a** antibiotic resistance classes, **b** MGEs, and **c** MRGs

In considering specific subcategories of resistance, the HFS sample contained the greatest relative abundances of beta lactamase, aminoglycoside, tetracycline, trimethoprim, fosfomycin, streptothricin, quinolone, and MLS antibiotic classes (Fig. 8). Note that these categories were also prominent in the WWTP and glacier samples, but to a lesser extent than in HIU and the LSS samples. In addition, although the multidrug category is highly abundant in HIU and LSS, it has the lowest relative abundance in the HFS sample. Interestingly, although HFS contained the highest relative abundance of total ARGs, the WWTP samples had the highest diversity of antibiotic resistance classes measured as the number of uniquely identified antibiotic types (Fig. 8). For instance, *sul1* was one of the most prevalent ARGs detected in WWTP samples [74]. However, *sul*1 was not found in the GEM sample. This is consistent with the *sul*1 gene being an anthropogenic marker of antibiotic resistance [75, 76]. Similarly, GEM has lower diversity of beta lactamase genes (4 beta lactamase ARGs) than the WWTP environments (25–237 beta lactamase ARGs). ARGs from acriflavine, triclosan, aminocoumarin, tetracenomycin, rifampin*,* and puromycin antibiotic classes were only detected in the WWTP and LSS samples. HK_INF and HK_AS indicated the highest relative abundance of ARGs compared to IND_AS and CHE_INF (Fig. 9a). Particularly, the HK_AS sample showed a decrease compared to HK_INF in the abundance of multidrug and aminoglycoside resistance genes, but an increase in the beta-lactamase*, MLS,* and trimethoprim antibiotic types.

### MGE abundance

For its MGE reference database, NanoARG curates a collection of genes related to mobility, including transposases, integrases, recombinases, and integrons, in addition to a curated database for the class 1 integron *intI*1 [64]. Transposases are the prominent MGEs across all samples (Fig. 9b). Interestingly, the HFS sample shows the lowest relative abundance of MGEs. The *Salmonella*-spiked sample along with the heavily infected urine sample shows a lower MGEs relative abundance compared to the environmental samples (WWTP and glacier). Note that the glacier sample, GEM, contained the lowest MGE abundance compared to the WWTP samples. Interestingly, GEM also has the lowest diversity of MGEs (integrases, transposases, and other MGEs) when compared to other samples. This suggests that there may be a lesser degree of HGT in relatively pristine environments, such as glaciers, than in heavily anthropogenically influenced environments, such as WWTPs. Further, the class 1 integron *int*I1, which has been proposed as an indicator of anthropogenic sources of antibiotic resistance [10], is also consistent with this trend. The integron *intI*1 was detected in all samples, except in the GEM sample, likely because glaciers are under less anthropogenic pressure such as antibiotics usage or wastewater discharges. In addition, *int*I1 in the HIU sample was ranked to be the highest in relative abundance, which is expected given the clinical context of this sample.

### MRG abundance

MRG profiles were markedly distinct when comparing trends among samples relative to ARG profiles. The HFS sample has the lowest number of MRGs, with only *merP* and *merT,* part of the mercury transport mechanism [62] (Fig. 9c). In contrast, LSS and HIU samples carried the highest relative abundance of MRGs. The lack of MRGs in HFS could be the result of the sample preparation and/or lack of direct selection pressures relevant to MRGs. Notably, the HFS sample carried high beta lactamase*,* aminoglycoside*,* tetracycline*,* and MLS abundance, contrasting with low multidrug relative abundance. WWTP samples showed a different trend compared to MGEs and ARGs. The CHE_INF sample has the lowest relative abundance of MRGs compared to other WWTP samples. Although CHE_INF has also the lowest ARG relative abundance, its MRG abundance was less than half that of any other WWTP sample, suggesting that the CHE_INF sample had less exposure to heavy metal compounds.

### Taxonomy profile

**Fig. 10:**
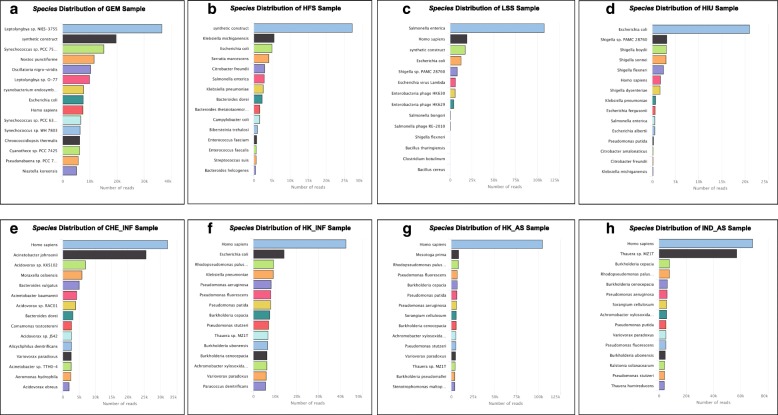
Taxonomic distribution of validation samples representing distinct biomes. **a** Phylum distribution of WWTP samples. **b**–**h** Bar plots with the total number of reads classified at the species taxonomy level for each validation sample

The HIU sample indicated *Escherichia coli* as the dominant species, which is expected given that a strain of MDR *E. coli* had been spiked into the urine prior to DNA extraction and analysis [43] (see Fig. 10d). Similarly, *Salmonella enterica* was found to be most abundant in the food sample metagenome (LSS), consistent with known *S. enterica* contamination of this sample [77]. The results of the HFS sample provide the opportunity to evaluate how the NanoARG taxonomic profiling performs with distinct approaches of library construction. Specifically, the HFS study [42] was designed to maximize chances of ARG detection, not to profile taxonomy. Thus, it makes sense that the nanopore taxonomy profile consists largely of *E. coli*, the expression host, and other taxa that likely represent the original source of the transformed ARGs, e.g., *Klebsiella pneumoniae*, *Serratia marcescens*, and *Enterococcus faecium* (see Fig. 10b). A surprise with respect to the species distribution in the WWTP samples was substantial detection of human DNA (see Fig. 10e–h). In one of the influent samples, *Homo sapiens* was the dominant species (see Fig. 10f–g). This host DNA is also observed to a lesser extent in the spiked samples (LSS, HIU). Surprisingly, the HFS sample did not contain detectable human DNA, suggesting that the technique employed in this study to specifically enrich ARGs during library preparation was successful for enriching ARGs.

### ARG neighboring gene analysis

**Fig. 11:**
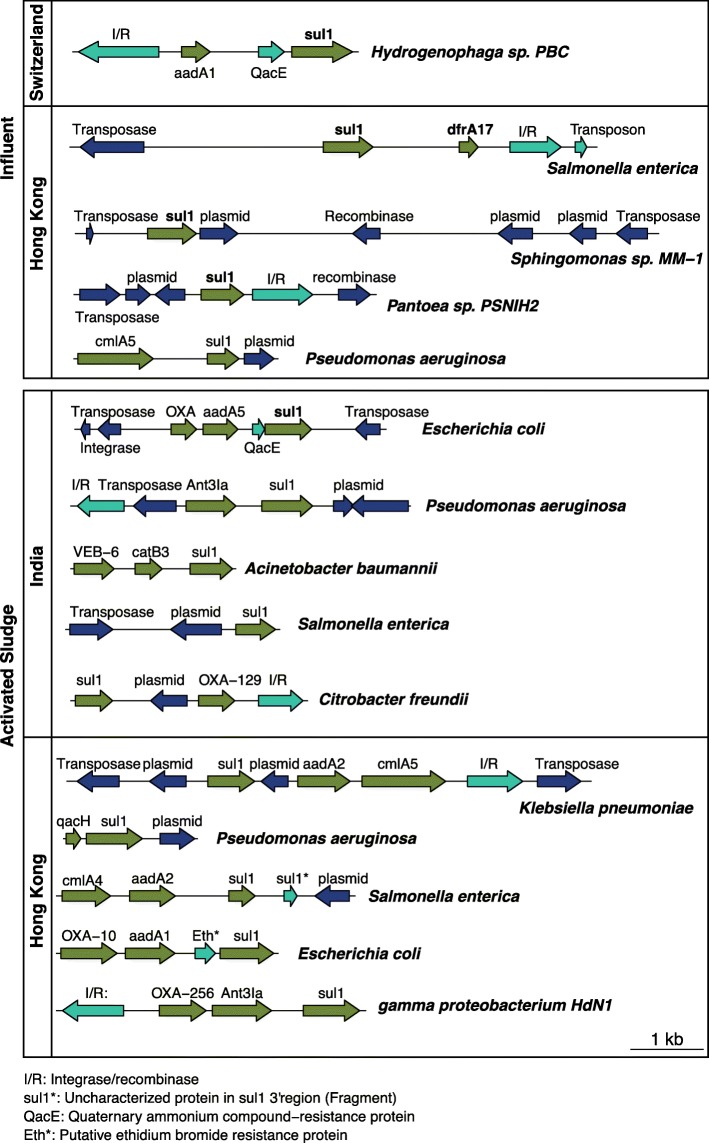
ARG patterns and contexts. Different patterns of ARGs for the WWTP samples (influent and activated sludge). I/R integrase/recombinase, *sul*1* uncharacterized protein in *sul*13’ region, *aqcE* quaternary ammonium compound-resistance protein, Eth* putative ethidium bromide resistance protein

Long nanopore sequences allow the inspection of ARG linkage patterns and the context of neighboring genes. For instance, Fig. 11 shows that the sulfonamide ARG *sul1* appears in different contexts depending on the WWTP sample and its host. Also, *sul1* is almost exclusively co-located together with *integrase*/*recombinase*, along with genes that have been found in plasmids, consistent with theory that *sul1* is an indicator of HGT. *sul1* was commonly observed together with an *integrase*/*recombinase* gene, followed by an aminoglycoside (*aadA*) gene, a determinant of quaternary ammonium compound resistance gene (*qacE*), which is also consistent with prevailing understanding of typical class 1 integron operon architecture [78]. Interestingly, this pattern seems to be modified in *E. coli* from two of the activated sludge environments (HK and IND), where the *integrase*/*recombinase* and the *aadA* region is interrupted by the insertion of a beta lactamase (*OXA*) gene. This linkage pattern differs from the one observed in *Hydrogenophaga* sp*. PBC* from the CHE influent*.* This *sul1* gene analysis is only one example of how NanoARG facilitates the inspection of colocation of ARG together with other genes of interest on the same DNA strand. Users can dig deeper to identify other patterns of interest and discover signals of ARG dissemination. The full co-occurrence result can be downloaded for further analysis.

**Fig. 12:**
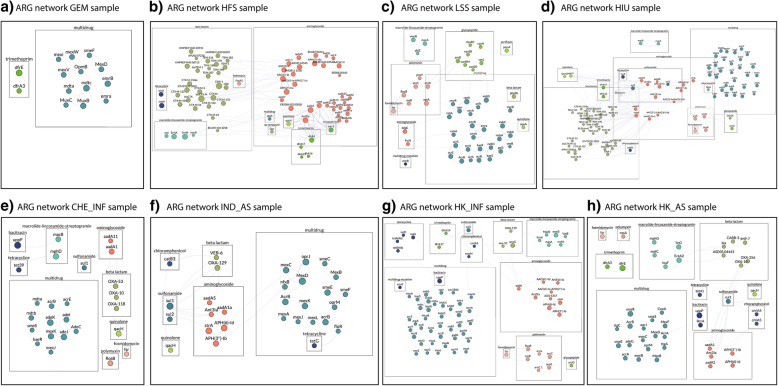
**a**–**h** ARG co-occurrence network for all samples

Figure 12 shows the ARG co-occurrence network for all samples. ARGs are linked if they co-occur within the same read and ARGs that appear only once are not shown. GEM, with a small number of ARGs belonging to only multidrug and trimethoprim classes, has no ARG co-occurrence (Fig. 12a). The WWTP samples show a common pattern of co-occurrence between beta-lactamases and aminoglycoside genes, indicating the high potential of these genes to be carried simultaneously. The HFS sample was dominated by aminoglycosides and beta lactamase genes, whereas LSS was dominated by multidrug genes and glycopeptide genes.

### Critical bacterial pathogens

Another important feature of NanoARG is the ability to putatively identify pathogens based on similarity to available DNA sequences in databases (see Table 2) and to assess their association with ARGs. For instance, DNA sequences corresponding to two of the three pathogens classified as having “critical importance” by the World Health Organization, *Acinetobacter baumannii* and *Pseudomonas aeruginosa*, were detected in all WWTP samples (see Table 4, Additional file 1: Table S1 and Additional file 2: Table S2). In contrast, DNA sequences corresponding to *Enterobacteriaceae* (carbapenem-resistant pathogen) were only detected in one WWTP sample (HK_INF). In addition, the HK_INF sample contained DNA sequences with high similarity to *Neisseria gonorrhoeae*. *Pseudomonas aeruginosa* was estimated to be the most abundant pathogen in the “critical” category across all samples and is particularly abundant in the IND_AS sample. No pathogen-like DNA sequences were found in the GEM sample, consistent with our expectation of a relative lack of anthropogenic influence. NanoARG clearly holds promise as a tool for screening for the potential presence of pathogens pertaining to various levels of priority. Further, the potential for putative pathogens to carry ARGs, MRGs, and MGEs can be readily assessed. However, it is important to emphasize that further culture-based and molecular-based analysis are required as follow-up to confirm the presence of viable and virulent pathogens.

**Table 4:** List of critically important bacterial pathogens putatively identified in the WWTP samples

Pathogen-like sequences	CHE_INF	IND_INF	HK_INF	HK_AS	GEM
*Acinetobacter baumannii*	3 (4)	4 (6)	12 (16)	6 (6)	0 (0)
*Pseudomonas aeruginosa*	7 (6)	58 (74)	12 (13)	7 (11)	0 (0)
*Enterobacteriaceae*	0 (0)	0 (0)	2 (2)	0 (0)	0 (0)
*Enterococcus faecium*	0 (0)	0 (0)	0 (0)	1 (1)	0 (0)
*Staphylococcus aureus*	0 (0)	0 (0)	0 (0)	1 (1)	0 (0)
*Helicobacter pylori*	0 (0)	0 (0)	0 (0)	0 (0)	0 (0)
*Campylobacter* spp.	0 (0)	0 (0)	0 (0)	1 (1)	0 (0)
*Salmonellae*	0 (0)	0 (0)	0 (0)	0 (0)	0 (0)
*Neisseria gonorrhoeae*	0 (0)	0 (0)	3 (6)	0 (0)	0 (0)
*Streptococcus pneumoniae*	0 (0)	0 (0)	1 (1)	0 (0)	0 (0)
*Haemophilus influenzae*	0 (0)	0 (0)	1 (1)	0 (0)	0 (0)
*Shigella* spp.	0 (0)	0 (0)	0 (0)	0 (0)	0 (0)

*Notation: number of reads (number of ARGs)

### NanoARG usage recommendation

Note that the various analyses provided by NanoARG are not restricted to nanopore sequencing reads. In fact, NanoARG can be applied to any set of long DNA sequences (> 1000 bp long). For instance, sequences from different technologies such as PacBio long-read sequencing or assembled contigs from short sequencing reads can be directly processed in NanoARG. Depending on specific research needs, different studies may have different requirements, e.g., some require more stringent criteria, whereas others are less. Thus, to allow for flexibility and customization, NanoARG provides users results produced by relaxed annotation parameters so that they can filter the results further to meet their specific needs. One caveat is that, because NanoARG uses the DeepARG-LS model to predict/detect ARGs, it inherits DeepARG’s limitation in that it cannot be used to identify ARGs whose resistance is conferred by SNPs or a small number of mutations from nonARGs [26]. For nanopore metagenomic data, because of the high error rate, it can be difficult to determine whether the differences in sequences are caused by real mutations or sequencing errors. Therefore, nanopore metagenomic sequencing might not be the ideal platform for identifying the ARGs that confer resistance through SNPs or a small number of mutations, unless a very high depth of coverages can be achieved.

## Conclusions

NanoARG is a public Web service dedicated to the analysis of ARGs from nanopore MinION metagenomes and is the first, to our knowledge, configured for analysis of environmental samples. While the platform was specifically developed for the analysis of environmental metagenomes generated from nanopore sequencing technologies, here we demonstrate that it also has broad potential for other types of data sets. As validated here using a combination of publicly available and in-house DNA sequence libraries, NanoARG can be used to profile ARGs in any biome, while also providing context of other co-located genes, such as MGEs, MRGs, and taxonomic markers. NanoARG provides a user-friendly interface for the analysis of any set of long DNA sequences (including assembled contigs), facilitating data processing, analysis, and visualization. Unlike other services dedicated exclusively to antimicrobial resistance (e.g., WIMP), NanoARG offers analysis of MRGs and MGEs while also enabling taxonomic annotation, identification of pathogen-like DNA sequences, and network analysis for assessing corresponding co-occurrence patterns. Further, integration with deep-learning based DeepARG facilitates a local strategy for annotating genes from long nanopore reads. Specifically, implementation of permissive parameters allows high flexibility for the detection of homologous genes, which helps overcome high error rate characteristic of nanopore sequences.

## Availability and requirements

NanoARG is a publicly available Web platform accessible at https://bench.cs.vt.edu/nanoarg. Users are required to create an account before uploading sequences to the platform. Finally, NanoARG accepts any type of long sequences in FASTA format.

## Additional files

Additional file 1: **Table S1.** *Pseudomonas aeruginosa*-like identified nanopore reads. (XLSX 4 kb)

Additional file 2: **Table S2.** *Acinetobacter baumannii-*like identified nanopore reads. (XLSX 4 kb)

Additional file 3: Nanopore sequencing data sets. **Figure S1.** The effect of sequencing error rates on the performance of NanoARG for the detection of ARGs. **Figure S2.** The effect of error rates on the performance of NanoARG for each antibiotic class. Figure S3. Effect of read length on the identification of ARGs. *Y*-axis is the success rate in identifying true ARGs. (DOCX 441 kb)

## Data Availability

NanoARG source code is available at https://bench.cs.vt.edu/nanoarg. Public datasets used in this study can be found at https://www.ebi.ac.uk/ena/data/view/PRJEB24565 (GEM), https://github.com/EvdH0/poreFUME (HFS), https://www.ncbi.nlm.nih.gov/sra?linkname=bioproject_sra_all&from_uid=352168 (HIU), and https://www.ncbi.nlm.nih.gov/bioproject/?term=PRJNA404022 (LSS). Wastewater treatment plant samples are under SRA bioproject PRJNA527877.
