# Supplementary material for: NanoARG: a web service for detecting and contextualizing antimicrobial resistance genes from nanopore-derived metagenomes
Source: Microbiome. 2019 Jun 7;7:88. doi: 10.1186/s40168-019-0703-9 (PMC6555988; doi:10.1186/s40168-019-0703-9)
Supplement: Supplementary file 3 — Nanopore sequencing data sets. Figure S1. The effect of sequencing error rates on the performance of NanoARG for the detection of ARGs. Figure S2. The effect of error rates on the performance of NanoARG for each antibiotic class. Figure S3. Effect of read length on the identification of ARGs. Y-axis is the success rate in identifying true ARGs. (DOCX 441 kb) [file 40168_2019_703_MOESM3_ESM.docx]

**NanoARG: A web service for detecting and contextualizing antimicrobial resistance genes from Nanopore-derived metagenomes**

G. A. Arango-Argoty^1^, D. Dai^2^, A. Pruden^2^, P. Vikesland^2^, L. S. Heath^1^, and L. Zhang^1*^

^1^Department of Computer Science, Virginia Tech, Blacksburg, VA, USA

^2^Department of Civil and Environmental Engineering, Virginia Tech, Blacksburg, VA, USA

* Corresponding author ([lqzhang@vt.edu)](about:blank)

# **Supplementary Material**

**Nanopore Sequencing Datasets**

**Heavily Infected Urine (HIU) Samples**

Publicly-available MinION data sets were obtained from heavily-infected urine downloaded from the European Nucleotide Archive (ENA) with the accession study number PRJEB16761 {Schmidt, 2017 #3015}. Briefly, this library consists of urine obtained from a healthy subject spiked with MDR *E. coli* and an *E. coli* strain cultivated from a heavily infected urine from the same study. The sample was sequenced within 48 hours. Details about the sampling methodology can be found in {Schmidt, 2017 #3015}.

**Arctic Glacier Extreme Metagenome (GEM)**

This data set was derived from a mixture of samples collected from Arctic cryoconite, located in an unnamed northern outlet glacier in central Svalbard {Edwards, 2016 #3152}. Nine samples were downloaded from the NCBI portal under the accession number PRJEB24565 and were combined into one large file that was further processed on the NanoARG Web service.

**Metagenomic Hospital Fecal Sample (HFS)**

This sample consists of DNA extracted from fecal samples of a patient treated with cephalosporins, flucloxacillin, bramycin (an aminoglycoside antibiotic), and colistin (a polymyxin antibiotic) during ICU care at the University Medical Center Utrecht in the Netherlands. Such treatment was carried out to treat the patient against gut colonization by nosocomial pathogens. Construction of the metagenomic library was done by a functional metagenomics approach that included a plasmid expression library supplemented with antibiotics, amplified with PCR, and sequenced with the MinION nanopore sequencer. Details about the sequencing protocol can be found in {van der Helm, 2017 #3153}.

**Metagenomic Lettuce Spiked *Salmonella* Sample (LSS)**

This library was constructed by inoculating *Salmonella* into food samples, including raw chicken breast, iceberg lettuce, black peppercorns, and peanut butter. The MinION nanopore sequencer was used to detect the presence of pathogens and was run for 1.5 hours after enrichment. Details about the library construction can be found at {Hyeon, 2018 #3154}.

**Effect of Sequencing Error Rate on ARG Detection**

The effect of different sequencing error rates on ARG detection was further tested by using a set of simulated datasets. DNA sequences were downloaded from the CARD database where those ARGs that confer resistance by single nucleotide polymorphisms (SNPs) were discarded. Duplicated sequences with 100% similarity were removed. Of the remaining 2,060 true ARGs, 50% were randomly selected (1,030). Then each selected ARG was mutated at random positions within its sequence reflecting or mimicing different error rates (5%, 10%, 15%, 20%, 25%, and 30%), and therefore a total of six datasets with different error rates were created. In addition to these true ARGs, a total of 1,686 genes from the human transcriptome, that is, nonARGs or true negatives, were randomly extracted and mutated at the same error rates as the true ARGs. Then ARGs and nonARGs were merged into one dataset for each error rate. To account for variation, this process was repeated five times for each one of the error rates. Finally, the simulated datasets were submitted to NanoARG website. The retrieved JSON files with the results were processed by custom scripts available at <https://github.com/gaarangoa/genomic-scripts>. Precision and recall were used to measure the rate of false positives and false negatives, respectively.

NanoARG achieved an overall precision of 0.99 and a recall of 0.98 across the six datasets (Supplementary Figure 1). Out of the 1,030 true ARGs, NanoARG predicted about 18 ARGs as nonARGs, and only an average of two nonARGs as ARGs. These results show that even though nanopore sequencing reads may have high sequencing errors, for an error rates below 30%, NanoARG is able to correctly differentiate ARGs from nonARGs. On the other hand, It is also important to see how good the performance is for different classes of ARGs. **Supplementary Figure** **2** shows the detailed classification for each ARG class. Note that antibiotic classes such as beta-lactam, aminoglycoside, diaminopyrimidine, fosfomycin, rifamycin had an almost perfect prediction independent of the error rate. Bacitracin and peptide have the lowest precision and recall. The overall precision is 0.98 and recall 0.97 for different error rates across different antibiotic classes.

**Effect of Read Length for Detecting ARGs**

To examine the effect of read length for the detection of ARGs, a mixture of bacterial genomes was built to generate a series of datasets of nanopore reads with a specific length. In detail, a set of 20 whole bacterial genomes were downloaded from the PATRIC database and submitted to NanoARG website. The true ARGs were defined as the hits with a strict 90% overlap to the reference ARGs and with a minimum of 80% sequence similarity. There were 14 unique true ARGs from the mixed genomes. For each genome in the sample, 1,000 reads with lengths of 1kb, 5kb, 10kb, 15kb and 20kb were selected at random positions within the genome of a fixed length. Thus, five read samples were generated with 1kb, 5kb, 10kb, 15kb and 20kb read lengths. The random sampling process was repeated five times considering the variability of the random sampling. For the simulated samples with different read lengths, we calculated the success rate, which is the proportion of true ARGs successfully identified in the simulated samples. A score of 1 means all ARGs were detected in the sample of the read length, whereas a score close to 0 means most of the ARGs were not detected in the sample of the read length. To compare true ARGs from identified ARGs, an identity of 80% was used as the similarity cutoff. As expected, the number of identified ARGs in the dataset of 1 kb read length was below 20%. This is not surprising as the dataset consists of only 1,000 reads selected at random from the whole genomes. When samples have a read length of 10 kb or greater, more than 60% of true ARGs can be detected (see **Supplementary Figure 3)**. It does not reach 100% given that only 1k reads were sampled. Results here show that having longer sequences could be more beneficial for ARG detection. Also longer sequences can contain more information so what context ARGs are in can also be inferred.


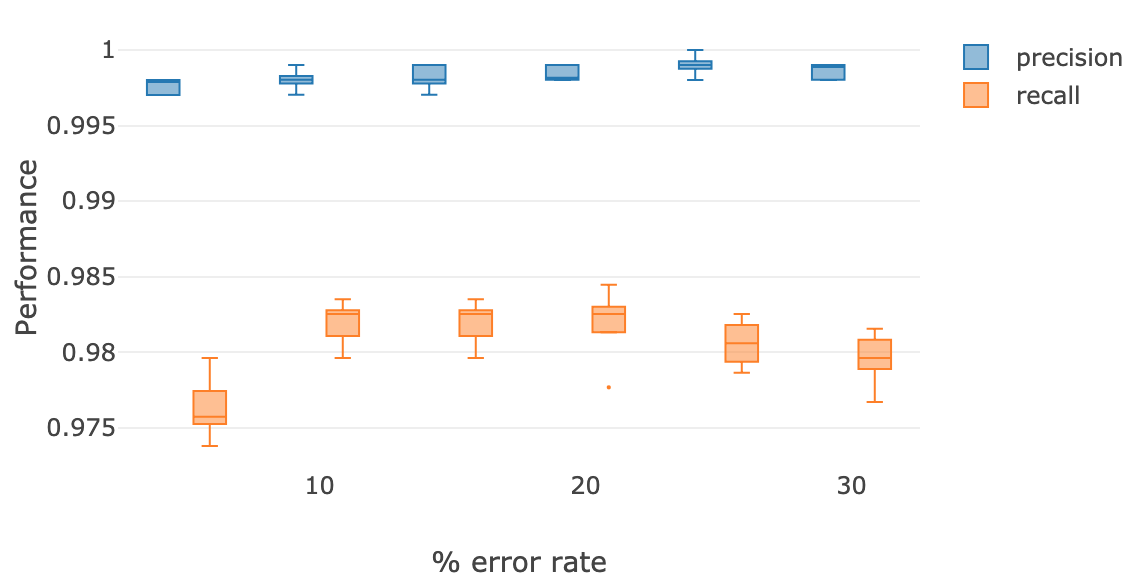


**Supplementary Figure 1**: The effect of sequencing error rates on the performance of NanoARG for the detection of ARGs.


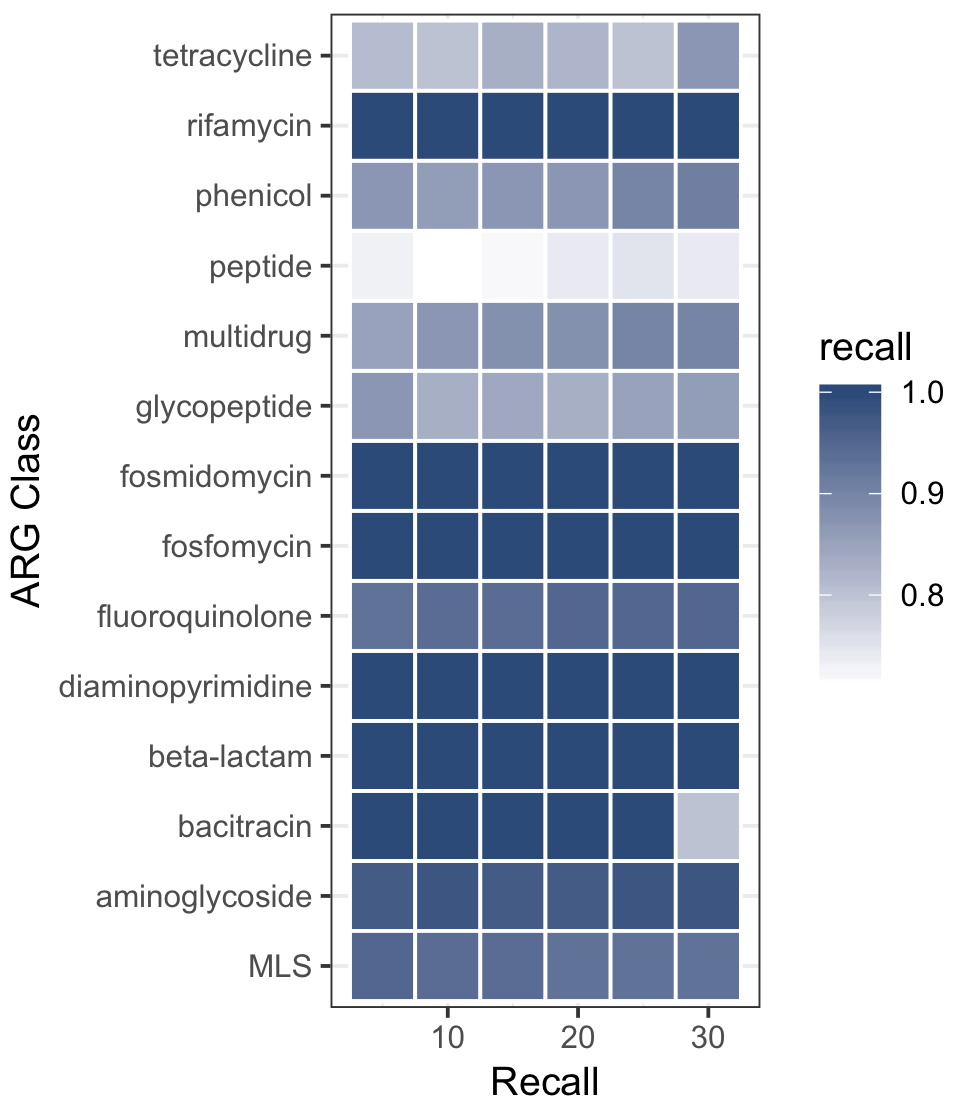


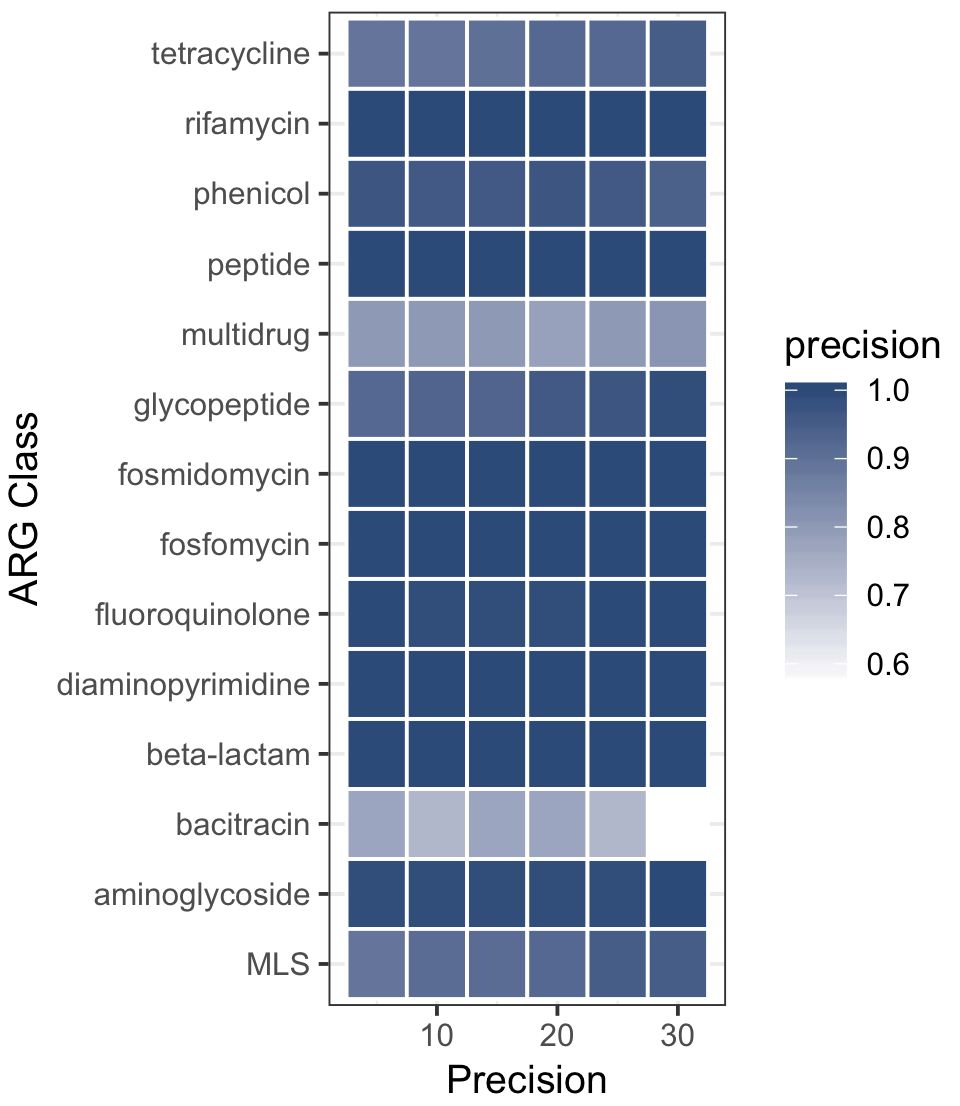

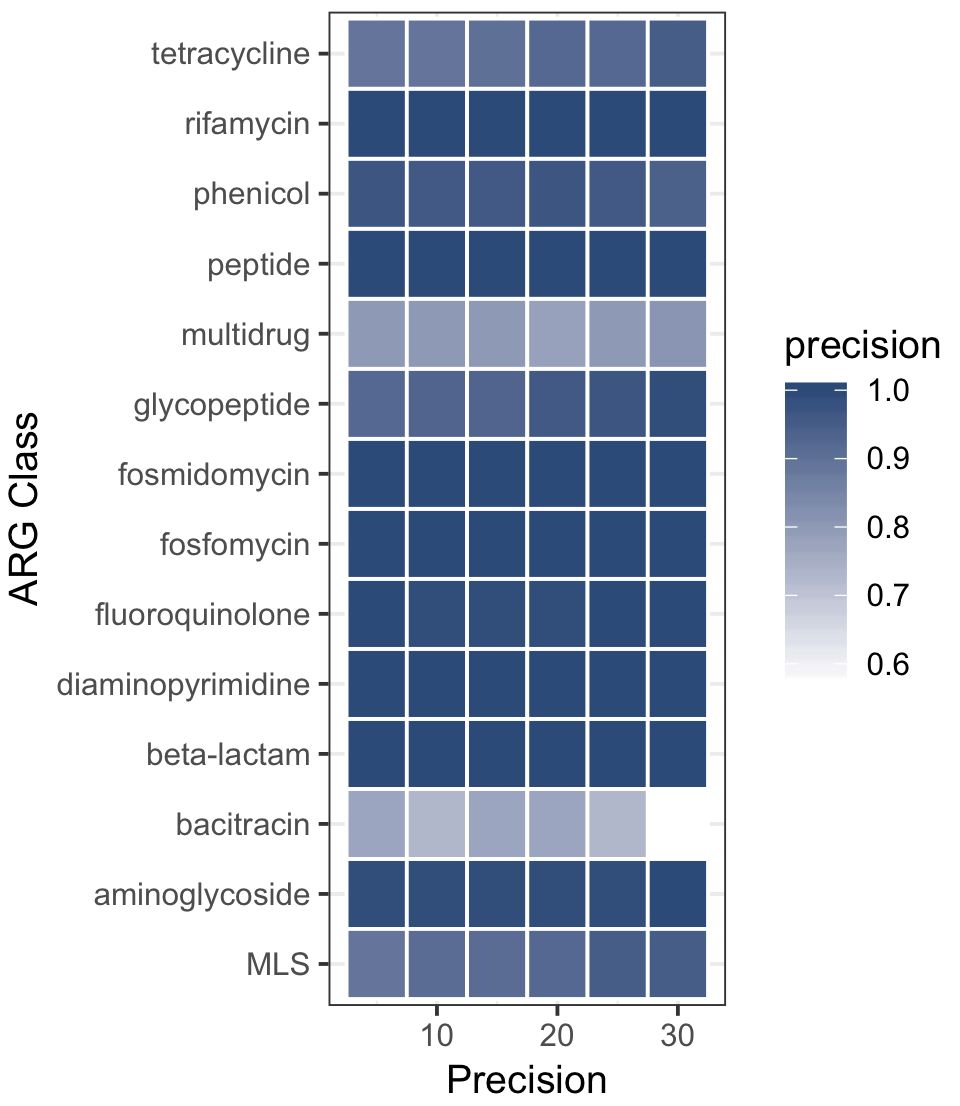


**Supplementary Figure 2:** The effect of error rates on the performance of NanoARG for each antibiotic class.


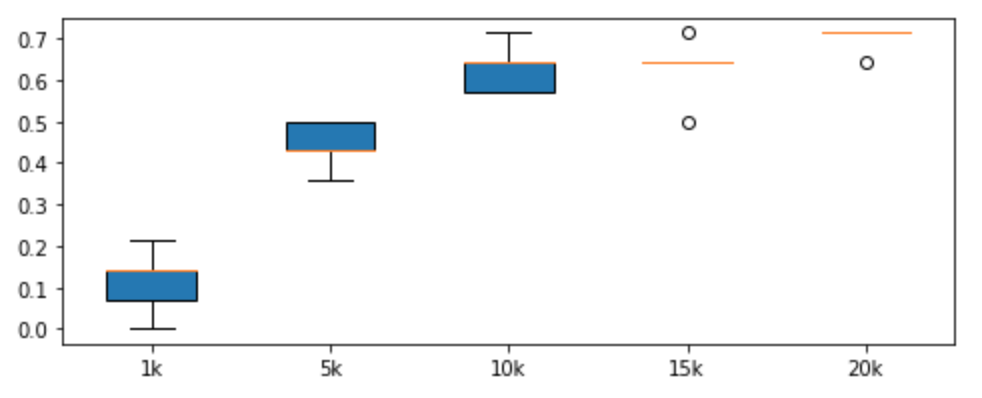


Read length (bp)

**Supplementary Figure 3:** Effect of read length on the identification of ARGs. Y-axis is the success rate in identifying true ARGs.
